# Oral 7,8‐Dihydroxyflavone Protects Retinal Ganglion Cells by Modulating the Gut‐Retina Axis and Inhibiting Ferroptosis via the Indoleacrylic Acid‐AhR‐ALDH1A3‐FSP1 Pathway

**DOI:** 10.1111/cns.70442

**Published:** 2025-05-14

**Authors:** Yanping Zhou, Yifan Feng, Yingxi Zhao, Yu Wu, Min Li, Xi Yang, Xinyuan Wu, Xiangwu Chen

**Affiliations:** ^1^ Department of Ophthalmology Zhongshan Hospital of Fudan University Shanghai China; ^2^ Department of Ophthalmology Eye Hospital of Wenzhou Medical University Wenzhou Zhejiang China; ^3^ Department of Ophthalmology Taizhou Hospital of Zhejiang Province Affiliated to Wenzhou Medical University Taizhou Zhejiang China

**Keywords:** 7,8‐Dihydroxyflavone, gut, Indoleacrylic acid, neuroprotection, TrkB

## Abstract

**Objectives:**

7,8‐Dihydroxyflavone (7,8‐DHF) activates the TrkB receptor, offering neuroprotection, yet its pharmacological limitations restrict its safe and effective delivery to the eye and brain, impeding clinical translation. This study explores the protective effects of oral 7,8‐DHF on retinal ganglion cells (RGCs) by inhibiting ferroptosis and investigates the involvement of the gut‐retina axis, particularly the Indoleacrylic acid (IDA)‐AhR‐ALDH1A3‐FSP1 pathway, with potential clinical implications.

**Methods:**

To evaluate the neuroprotective effects of oral 7,8‐DHF, retinal 3D cultures were used for axon regeneration and GCL cell apoptosis, and ONC models for RGC survival and electrophysiology. Mechanisms were investigated by assessing ferroptosis‐related proteins via Western blotting, screening differential metabolites in PC12 cells, analyzing mitochondrial changes with TEM, evaluating gut microbiota shifts, and examining metabolite changes in retina and feces.

**Results:**

Oral 7,8‐DHF enhanced RGC survival and retinal function in the ONC model by inhibiting ferroptosis, independent of TrkB activation. This effect was blocked by antibiotics and AHR, ALDH1A3, and FSP1 inhibitors. Metabolomics showed increased IDA in retina and feces, with IDA inhibiting ferroptosis in PC12 cells and promoting axonal regeneration in retinal explants. Western blot revealed upregulation of nAhR and ALDH1A3, while non‐FSP1 ferroptosis proteins were unaffected. 7,8‐DHF also altered gut microbiota, increasing Parasutterella, which correlated with higher IDA levels.

**Conclusions:**

7,8‐DHF regulates the gut microbiota to increase IDA levels in the intestine, which subsequently leads to the accumulation of IDA in the retina. This activates the AhR‐ALDH1A3‐FSP1 axis in the retina, thereby inhibiting retinal ferroptosis and exerting neuroprotective effects.

## Graphical abstract

Oral 7,8‐Dihydroxyflavone (7,8‐DHF) protects retinal ganglion cells by modulating the gut‐retina axis, increasing indoleacrylic acid (IDA) levels, and inhibiting ferroptosis via the AhR‐ALDH1A3‐FSP1 pathway, offering a novel therapeutic strategy for retinal neuroprotection.

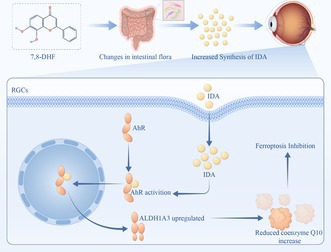

**Abbreviations**
16‐B1P: 16‐B1‐Phytopropanol
7,8‐DHF: 7,8‐Dihydroxyflavone
AhR: Aryl hydrocarbon Receptor
BDNF: Brain‐Derived Neurotrophic Factor
CoQ10: Coenzyme Q10
IDA: Indoleacrylic Acid
NA: Nonadecylic Acid
OD: Optical Density
ONC: Optic nerve crush
RGCs: Retinal Ganglion Cells
SCFA: Short‐Chain Fatty Acid
TBA: 4‐Trimethylammoniobutanoic Acid
TEM: Transmission Electron Microscopy
TrkB: Tropomyosin Receptor Kinase B

## 1 Introduction

Optic neurodegenerative diseases like glaucoma cause irreversible vision loss due to progressive retinal ganglion cell (RGC) degeneration [1, 2]. While molecular insights have advanced, effective therapies are still lacking [3]. 7,8‐Dihydroxyflavone (7,8‐DHF), a natural flavonoid and selective TrkB agonist, offers neuroprotection [4, 5]. However, its clinical use is limited by poor pharmacokinetics, including rapid metabolism and low retinal and plasma levels [4, 5].

Recent studies highlight the gut‐retina axis's role in retinal health, with gut microbiota influencing retinal diseases via immune and metabolic interactions [6]. Tryptophan‐derived indole compounds, like indoleacrylic acid (IDA), show neuroprotective effects through the aryl hydrocarbon receptor (AhR) pathway [7, 8].

This study identifies a novel mechanism of 7,8‐DHF neuroprotection in retinal neurons. Oral 7,8‐DHF exerts its effects through gut‐retina axis modulation, increasing IDA levels that inhibit retinal ferroptosis via the AhR‐ALDH1A3‐FSP1 pathway. This suggests a new therapeutic approach for neurodegenerative diseases, addressing the challenges in 7,8‐DHF's clinical application.

## 2 Materials and Methods

### 2.1 Ethics Statement

The research was approved by the Animal Research and Ethics Committee at Zhongshan Hospital, Fudan University, and complied with NIH and ARRIVE guidelines for ethical animal treatment.

### 2.2 Retinal Three‐Dimensional (3D) Culture

On postnatal day 3, C57BL mice (Shanghai Jiesijie Laboratory Animal Co., LTD) were euthanized with sodium pentobarbital, and retinas were dissected. The explants were exposed in 0.3% type I collagen solution, containing 10× MEM, NaOH, NaHCO3, and HEPES (8:1:1) and incubated at 37°C for 10 min, followed by incubation in MEM with NaHCO3, glucose, HEPES, putrescine, penicillin–streptomycin, Na_2_SeO_3_, and FBS at 37°C with 5% CO_2_, as per Chen et al. [9].

### 2.3 Immunohistochemical Analysis of Neurite Extension in Retinal Explants

Retinal explants (day 6) were fixed in paraformaldehyde, washed in PBS, permeabilized with Triton X‐100, and incubated in goat serum. Overnight at 4°C, explants were treated with mouse anti‐Thy1.1 (#14‐0900‐81, Invitrogen, USA) and rabbit anti‐GAP‐43 (#PA5‐79299, Invitrogen, USA). Post‐incubation, explants were rinsed and incubated with FITC‐ and Cy3‐conjugated secondary antibodies (#A24538, Invitrogen, USA; #M30010, Invitrogen, USA). Neurite extension was imaged using a DMI 3000B fluorescence microscope (Leica, Germany) at 480 nm (FITC) and 546 nm (Cy3).

### 2.4 Evaluation of Neurite Regeneration in Retinal Explants

Six experimental groups were created: a control group with 1‰ DMSO in the culture medium, a group with Fer‐1 (1 μM), a group with IDA (10 μM; #I2273, Sigma, CA, USA), a group with both CH‐223191 (iAhR; 5 μM; #C8124, Sigma, CA, USA) and IDA (10 μM), a group with both DEAB (iALDH1A3; 10 μM; #D86256, Sigma, CA, USA) and IDA (10 μM), and a group with both iFSP1 (1 μM; #150651–39‐1, Yeasen, Shanghai, China) and IDA (10 μM). The density and length of neurite outgrowth were evaluated according to Chen et al. [9].

### 2.5 TUNEL Staining

After three days of culture, retinal explants were fixed with paraformaldehyde, cut into 10 μm slices, and stained using a TUNEL kit (#C1088, Beyotime, Shanghai, China). Sections were co‐stained with DAPI and observed under a fluorescence microscope. The apoptotic rate in the GCL was determined by the ratio of TUNEL‐positive to DAPI‐positive cells. Eight sections from six explants per group were evaluated.

### 2.6 Optic Nerve Crush (ONC) Mouse Model

C57/BL mice received 50 mg/kg sodium pentobarbital intraperitoneally and 0.5% proparacaine hydrochloride topically. A conjunctival peritomy exposed the optic nerve, accessed with dilating forceps. Dumont #5 forceps (FST, Heidelberg, Germany) clamped the optic nerve 1 mm behind the optic disk for 5 s to induce crush injury. Mice with severe bleeding or unrecovered retinal circulation within 5 min were excluded.

### 2.7 Counting Analysis of RBPMS‐Positive Cells

To assess 7,8‐DHF on RGC survival, mice were gavaged daily for 14 days pre‐surgery. Sham and ONC groups received PBS with sham or ONC surgery. The ONC + 10, 25, or 50 mg/kg 7,8‐DHF groups received corresponding doses pre‐ONC.

For antibiotics, IDA, iAhR, iALDH1A3, and iFSP1 effects on RGC survival, C57BL/6J mice received intravitreal injections of iAhR (10 μM, 1 μL), iALDH1A3 (20 μM, 1 μL), iFSP1 (2 μM, 1 μL), or vehicle before daily gavage with 25 mg/kg 7,8‐DHF or PBS for 14 days. On day 15, a second intravitreal injection preceded sham or ONC: Sham (sham surgery, PBS), ONC (ONC surgery, PBS), ONC +7,8‐DHF (ONC, 7,8‐DHF), ONC +7,8‐DHF + Abx (ONC, 7,8‐DHF, antibiotics in water), ONC +7,8‐DHF + Abx + IDA (ONC, 7,8‐DHF, antibiotics, daily 20 mg/kg IDA), and ONC +7,8‐DHF + iAhR/iALDH1A3/iFSP1 (ONC, 7,8‐DHF, intravitreal iAhR, iALDH1A3, or iFSP1). Treatments continued post‐surgery for two weeks.

Two weeks post‐ONC, mice were euthanized for retinal fixation and RGC quantification via anti‐RBPMS (1:1000; CA# PA5‐31231, Invitrogen, USA) immunostaining. Whole‐mount immunohistochemistry assessed RBPMS‐positive cells in eight retinal regions, with images taken 200 μm from the periphery. A blinded observer counted cells using ImageJ's plugin.

### 2.8 Electroretinogram Recordings

The mice were divided into two groups: ONC (PBS gavage) and ONC +7,8‐DHF (25 mg/kg gavage). To examine the effects of antibiotics, IDA, iAhR, iALDH1A3, and iFSP1 on PhNR amplitude, the same grouping structure was used.

Retinal function was assessed using full‐field flash electroretinography (HMsERG system; OcuScience, NV, USA). Recording electrodes were placed on the dilated, anesthetized eyes. Scotopic flashes at 0.1 cd/m^2^ were administered, and data were recorded. ERGView 4.380R software with a 150 Hz low‐pass filter processed the data. PhNR amplitude was measured from the lowest point relative to baseline.

### 2.9 Transmission Electron Microscopy (TEM)

Mice were grouped into ONC (PBS gavage) and ONC +7,8‐DHF (25 mg/kg gavage). Retinal electron microscopy was conducted two weeks post‐surgery (three mice per group), analyzing eight fields per eye. Retinal sections (100 μm) were fixed, dehydrated, embedded in Durcupan ACM, sectioned (0.1 μm), stained with 3% lead citrate, and examined via transmission electron microscopy (Carl Zeiss, Oberkochen, Germany).

### 2.10 PC12 Cell Culture and Treatment

PC12 cells were cultured in PC12‐specific medium obtained from Beyotime Biotechnology, supplemented with 1% FBS and 1% penicillin–streptomycin, and maintained at 37°C in a humidified atmosphere with 5% CO_2_.

Cells were treated with the ferroptosis inducer RSL3 (100 nM) and test compounds. IDA was added at 0.01, 0.1, 1, 10, and 100 μM. Other groups received Nonadecylic Acid (NA; #N5252, Sigma, USA), 16‐B1‐Phytop (Yuhao Chemical, Hangzhou, China), or 4‐Trimethylammoniobutanoic Acid (TA; #BCN1744, BioCrick, Chengdu, China) at 0.05–500 μM (NA, 16‐B1‐Phytop) or 0.1–100 mM (TA). Control cells were treated with DMSO. All treatments were added to the medium with Fer‐1 for simultaneous exposure to the inducer and test compound.

Following 24 h of treatment, cells were harvested for cell viability assays and protein expression analysis by Western blotting. For protein expression analysis, cells were lysed in RIPA buffer containing protease and phosphatase inhibitors. Protein concentrations were determined using the BCA assay, and samples were prepared for Western blot analysis following standard protocols.

### 2.11 Cell Viability Assay

Cell viability was measured using CCK8 assays. Cells (3000 per well) were seeded in 96‐well plates, treated with drugs for 24 h, and 10 μL of CCK8 reagent was added for 3 h. The optical density (OD) was measured at 450 nm.

### 2.12 Microbial Diversity

Fecal samples from the ONC and ONC + 25 mg/kg 7,8‐DHF groups were analyzed for microbial diversity. DNA was extracted, and the V3–V4 region of the 16S rRNA gene was PCR amplified. Amplicons were quantified, pooled, and sequenced on the Illumina MiSeq platform. DADA2 processed the quality‐filtered reads to generate sequence variants. Alpha diversity was assessed using Chao1 and Shannon indices, and beta diversity by NMDS and hierarchical clustering. LEfSe identified significant taxa, and LDA scores evaluated effect sizes. Standard bioinformatics pipelines ensured data consistency.

### 2.13 Western Blot

Western blotting with customized antibody preparations followed a consistent protocol and buffer setup. TrkB phosphorylation at Y816, Y515, and Y706 in ONC mouse retinas was analyzed at 12, 24, and 48 h post‐7,8‐DHF treatment using antibodies: rabbit anti‐pY816‐TrkB (1:1000, #ABN1381, Sigma), rabbit anti‐pY515‐TrkB (1:1000, #SAB4503785, Sigma), rabbit anti‐pY706‐TrkB (1:1000, #SAB4300702, Sigma), goat anti‐TrkB (0.1 μg/mL, #AF1494, R&D Systems), and rabbit anti‐β‐actin (1:2000, #AF5003, Beyotime). Intracellular levels of nrf2, NQO1, SLC7A11, and AhR in PC12 cells post‐IDA exposure were measured using rabbit anti‐nrf2 (1:1000, #SAB4501984, Sigma), rabbit anti‐NQO1 (1:1000, #SAB5701284, Sigma), rabbit anti‐SLC7A11 (1:1000, #SAB5700735, Sigma), and rabbit anti‐AhR (1:1000, #SAB4500725, Sigma). IDA's impact on ferroptosis proteins and AhR, ALDH1A3, and FSP1 inhibitors was examined in vitro and in vivo using rabbit anti‐AhR (1:1000, #SAB4500725, Sigma), rabbit anti‐ALDH1A3 (1:1000, #ABN427, Sigma), rabbit anti‐FSP1 (1:1000, #A06541‐2, Boster), rabbit anti‐GPX4 (1:1000, #SAB5700944, Sigma), rabbit anti‐ACSL4 (1:2000, #SAB2100035, Sigma), rabbit anti‐SLC7A11 (1:1000, #SAB5700735, Sigma), rabbit anti‐GCH1 (1:1000, #PA5‐103865, Invitrogen), rabbit anti‐FTH1 (1:500, #ZRB2695, Sigma), rabbit anti‐DHODH (1:1000, #SAB2100574, Sigma), rabbit anti‐4‐HNE (1:1000, #MA5‐27570, Invitrogen), and rabbit anti‐β‐actin (1:2000, #AF5003, Beyotime).

Mice were euthanized, and retinas stored at −80°C. Proteins from retinal and PC12 cells were extracted using RIPA buffer with inhibitors. Nuclear proteins, including AhR, were isolated using a nuclear extraction kit (Beyotime, Shanghai, China). Protein concentrations were measured via BCA assay (Beyotime, Shanghai, China). Proteins were separated by 12% SDS‐PAGE and transferred to PVDF membranes (Thermo Fisher Scientific, MA, USA). After 1‐h blocking with 5% skim milk, membranes were incubated with primary antibodies overnight at 4°C. After TBST washes, membranes were incubated with HRP‐conjugated secondary antibodies (#32460, #31430, #31402, Invitrogen, CA, USA) for 1 h. Blots were detected using the Enhanced Chemiluminescence System (Amersham Biosciences, NJ, USA), and band intensity was quantified with ImageJ. Western blotting was done in triplicates per group.

### 2.14 Metabolomic Analysis

ONC mice were divided into ONC, ONC +7,8‐DHF, and ONC +7,8‐DHF + Abx groups. Fecal and retinal samples from C57BL/6 mice, each with twelve pooled retinas (six per group), were stored at −80°C. A 60 mg sample was homogenized with internal standards in a 4:1 methanol–water mixture, incubated at −20°C, filtered, centrifuged, and stored at −80°C for LC–MS analysis. Metabolic profiling used an ACQUITY UPLC I‐Class Plus, Q Exactive mass spectrometer, and ACQUITY UPLC HSS T3 column. The gradient was 98% A (0–2 min), linear to 2% A (2–11 min), then re‐equilibrated at 98% A (11–13 min). Column temperature was 45°C, with 2 μL injection at 0.35 mL/min. Mass spectrometry data (m/z 100–1500) were acquired at 17,500 resolution, with 10, 20, and 40 eV for MS/MS spectra and a spray voltage of 3.5 kV.

### 2.15 Statistical Analysis

Statistical analyses were performed using SPSS 22.0. Normality was assessed with the Shapiro–Wilk test. For normally distributed data (*p* > 0.05), two‐group comparisons were performed using Student's *t*‐test, or Welch's *t*‐test when variances were unequal (Levene's test). For non‐normal data (*p* ≤ 0.05), the Mann–Whitney *U* test was used. Data are presented as mean ± SEM, with *p* < 0.05 considered statistically significant. For analyses involving multiple comparisons, Benjamini‐Hochberg (BH) correction was applied to control the false discovery rate (FDR), and adjusted *p*‐values are reported where applicable. MaAslin analysis assessed the relationship between microbial community abundance and metabolite relative abundance, adjusting for 7,8‐DHF treatment, age, and weight. No formal statistical power analysis was conducted.

## 3 Results

### 3.1 Oral Administration of 7,8‐DHF Protects RGCs by Inhibiting Retinal Ferroptosis Without Activating Retinal TrkB Receptor

**FIGURE 1:**
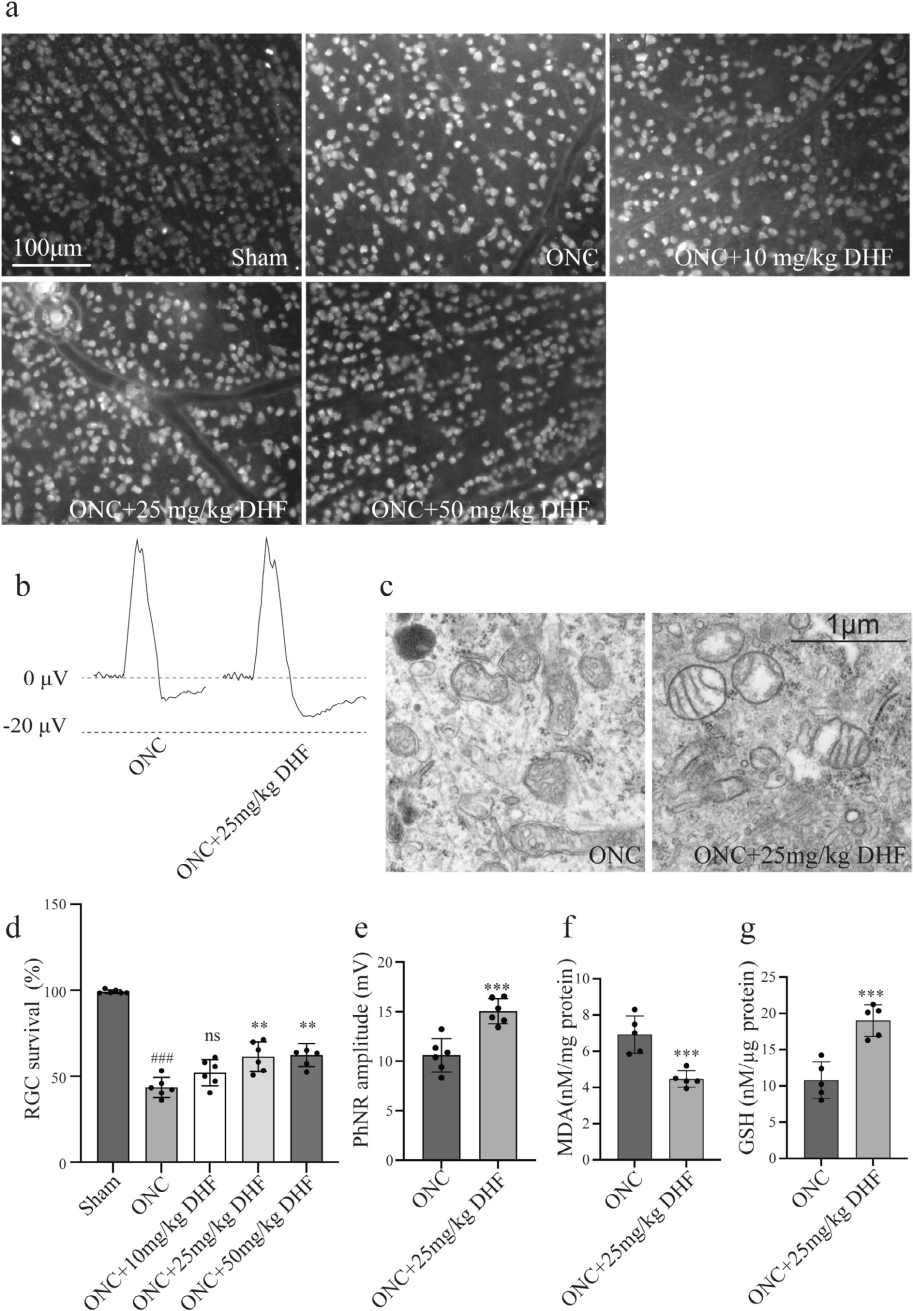
Effects of 7,8‐DHF on RGC survival, retinal electrophysiology, and oxidative stress after ONC. (a) RBPMS immunofluorescence staining of retinal flat mounts from sham, ONC, and ONC +7,8‐DHF groups (10, 25, and 50 mg/kg). Scale bar: 100 μm. (b) PhNR waveforms showing electrophysiological changes in ONC and ONC +25 mg/kg 7,8‐DHF groups. (c) TEM images of retinal sections showing mitochondrial morphology in ONC and ONC +25 mg/kg 7,8‐DHF groups. Scale bar: 1 μm. The ONC group displays damaged mitochondria, while the 7,8‐DHF group shows more intact mitochondria. (d) Quantitative analysis of RGC survival rates. 7,8‐DHF at 25 mg/kg and 50 mg/kg significantly improved survival, while 10 mg/kg showed no significant difference. (e) Quantification of PhNR amplitude in ONC +25 mg/kg 7,8‐DHF group. (f, g) MDA levels (f) were significantly reduced, and GSH levels (g) significantly increased in ONC +25 mg/kg 7,8‐DHF compared to ONC. Data are presented as mean ± SEM. *n* = 6 for (d, e); *n* = 5 for (f, g). Mann–Whitney *U* test was used for Sham vs. ONC in (d); other comparisons used unpaired *t*‐test. ###*p* < 0.001 vs. sham; **p < 0.01, ***p < 0.001 vs. ONC; ns, *p* > 0.05 vs. ONC.

The ONC group had significantly reduced RGC survival compared to the sham group (43 ± 5.76 vs. 99.17 ± 1.09, *p* < 0.001) (Figure 1a,d). Treatment with 25 mg/kg and 50 mg/kg 7,8‐DHF significantly improved survival (61.47 ± 8.64 vs. 43.50 ± 5.76, *p* < 0.01; 62.33 ± 6.77 vs. 43.50 ± 5.76, *p* < 0.01), while 10 mg/kg showed no significant difference (52.06 ± 7.72 vs. 43.50 ± 5.76, *p* > 0.05). A 25 mg/kg dose of 7,8‐DHF demonstrated effective neuroprotection.

The ONC +25 mg/kg 7,8‐DHF group had a significant increase in PhNR amplitude (15.04 ± 1.27 vs. 10.59 ± 1.69, *p* < 0.001; Figure 1b,e).

TEM images of retinal sections revealed that the ONC group had numerous shrunken mitochondria, whereas the ONC + 25 mg/kg 7,8‐DHF group displayed fewer shrunken mitochondria and more normal mitochondria (Figure 1c).

Retinal MDA levels were lower (6.93 ± 1.03 vs. 4.47 ± 0.46, *p* < 0.001) and GSH levels were higher (10.80 ± 2.51 vs. 18.99 ± 2.20, *p* < 0.001) in the ONC + 25 mg/kg 7,8‐DHF group compared to the ONC group (Figure 1f,g). No significant differences in retinal TrkB phosphorylation or total TrkB protein levels were detected between groups (Figure S1a–e).

### 3.2 Oral Administration of 7,8‐DHF Upregulates Gut IDA Levels, Which Exhibit Ferroptosis Inhibition Effects

**FIGURE 2:**
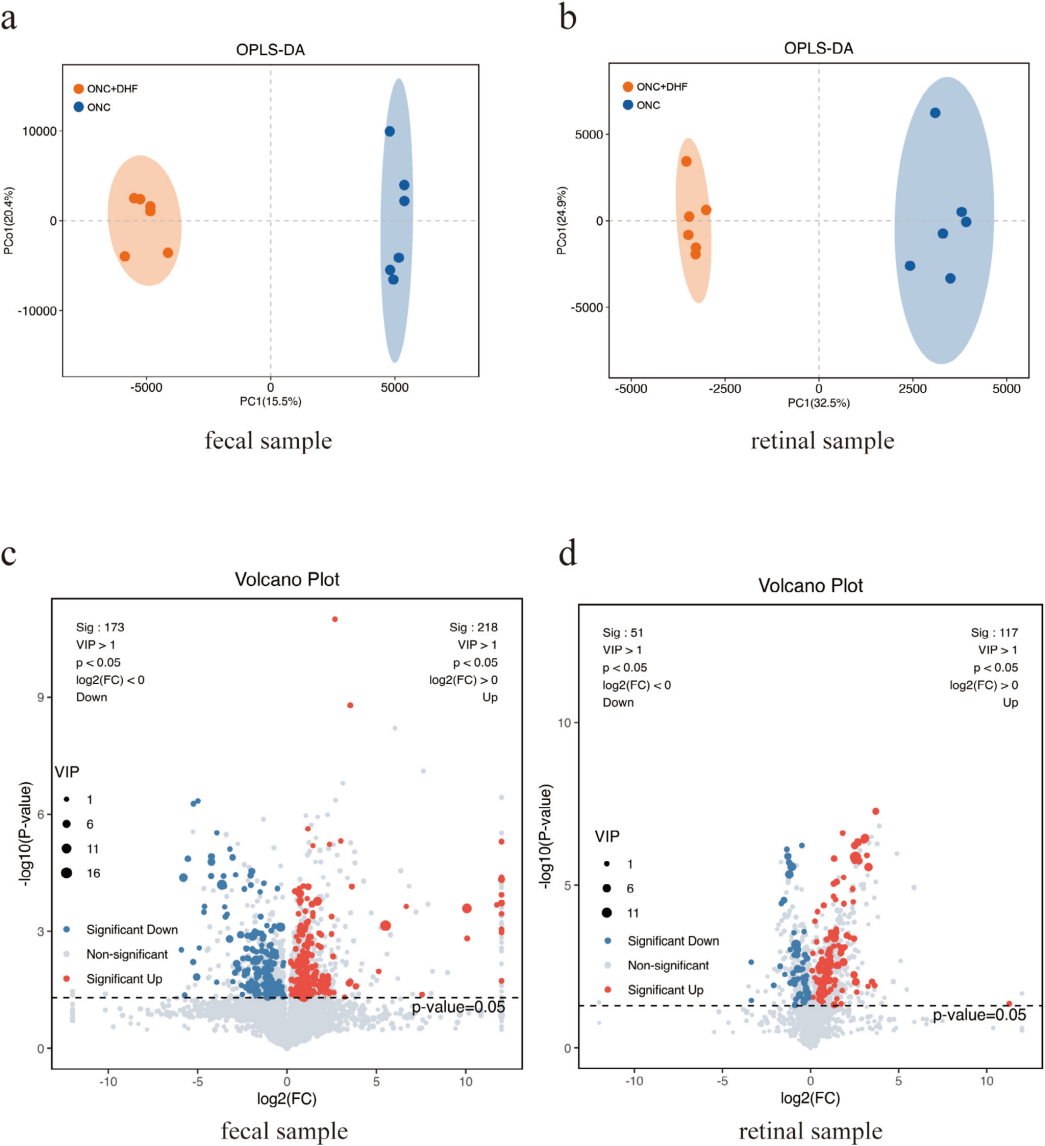
Phosphorylation and expression levels of TrkB in the retina treated by 7,8‐DHF following ONC injury. (a) Representative Western blot images showing phosphorylation of TrkB at Y515, Y816, and Y706, along with total TrkB and β‐actin expression, at 12 h, 24 h, and 48 h following 7,8‐DHF administration in ONC‐induced retinal injury. (b–e) Quantitative analysis of TrkB phosphorylation at Y816 (b), Y706 (c), and Y515 (d), normalized to total TrkB, and total TrkB expression normalized to β‐actin (e). No significant differences were observed in phosphorylation or total TrkB expression between the ONC +7,8‐DHF and ONC groups at any time point. Data are presented as mean ± SEM; unpaired *t*‐test for panels (b‐e), all *p* > 0.05; *n* = 3 for all panels.

OPLS‐DA analyses of both fecal and retinal metabolomics (Figure 2a,b) revealed distinct metabolic profiles between ONC and ONC + 7,8‐DHF groups. In fecal samples, 391 differential metabolites were identified (VIP > 1, *p* < 0.05; Figure 2c), including 218 upregulated and 173 downregulated. Similarly, retinal metabolomics (Figure 2d) revealed 168 differential metabolites, with 117 upregulated and 51 downregulated.

**FIGURE 3:**
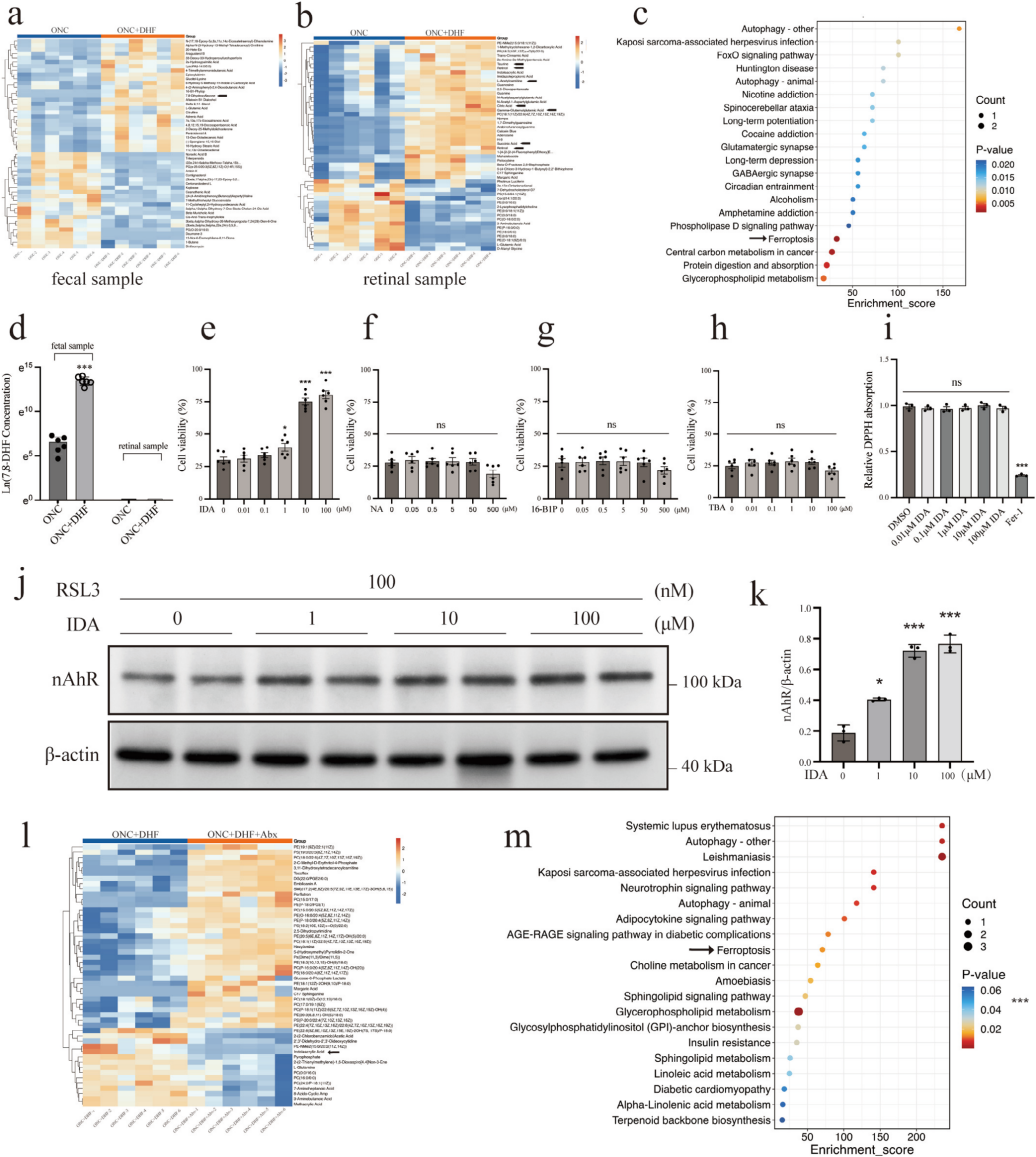
Metabolic effects of 7,8‐DHF on fecal and retinal samples in ONC mice, and the impact of IDA and other metabolites on ferroptosis and associated mechanisms in PC12 cells and retinal tissues. (a) Heatmap of the top 50 differential metabolites in fecal samples showing significantly elevated 7,8‐DHF levels in the ONC +7,8‐DHF group compared to ONC. (b) Heatmap of the top 50 differential metabolites in retinal samples highlighting increased ferroptosis‐inhibiting metabolites, including taurine, retinal, retinol, L‐acetylcarnitine, citric acid, gamma‐glutamylglutamic acid, and succinic acid, in the ONC +7,8‐DHF group. (c) Pathway enrichment analysis of retinal metabolites showing significant downregulation of ferroptosis pathways in the ONC +7,8‐DHF group, suggesting a regulatory effect of 7,8‐DHF on ferroptosis‐related pathways. Arrowheads and arrows indicate key metabolites. (d) Ln‐transformed 7,8‐DHF levels showing a significant increase in fecal samples of the ONC +7,8‐DHF group with no detectable levels in retinal samples. (e) Dose‐dependent effects of IDA (0–100 μM) on cell viability in RSL3 (100 nM)‐induced ferroptosis in PC12 cells. IDA significantly increased viability at 1, 10, and 100 μM. (f–h) Effects of NA, 16‐B1P, and TBA on cell viability in RSL3 (100 nM)‐induced ferroptosis. No significant effects were observed at any concentration. (i) DPPH assay showing antioxidant capacity of IDA (0.01–100 μM). IDA did not reduce DPPH absorption, while Fer‐1 significantly decreased absorption. (j) Western blot analysis of nAhR in PC12 cells treated with RSL3 and IDA (0–100 μM). (k) Quantification of nAhR protein level. IDA significantly increased nAhR expression at 1, 10, and 100 μM. (l) Heatmap showing reduced IDA levels in ONC + DHF + Abx vs. ONC + DHF groups. (m) Pathway enrichment analysis of differentially expressed metabolites in ONC + DHF + Abx group, highlighting ferroptosis‐associated pathways. Data are mean ± SEM (*n* = 6 for a‐h, l, m; *n* = 3 for i, k). Unpaired *t*‐test for (d, e, k); one‐way ANOVA with Tukey's post hoc test for (f‐i). *p < 0.05, ***p < 0.001 vs. control; ns (above horizontal lines), *p* > 0.05 among groups below the line.

Heatmaps of the top 50 differential metabolites in feces (Figure 3a) showed a significant increase in 7,8‐DHF levels in the ONC + 7,8‐DHF group vs. the ONC group (6.24 ± 0.98 vs. 13.46 ± 0.48, *p* < 0.001; Figure 3d). In retinal samples, the heatmap (Figure 3b) revealed elevated levels of ferroptosis‐inhibiting metabolites, such as taurine, retinal, retinol, L‐acetylcarnitine, citric acid, gamma‐glutamylglutamic acid, and succinic acid. The compound 7,8‐DHF itself was undetected in retinal tissue (Figure 3d).

Pathway enrichment analysis of differential metabolites in retinal samples (Figure 3c) revealed downregulation of ferroptosis pathways in the ONC + 7,8‐DHF group compared to the ONC group. From the overlap of significantly increased metabolites in both retinal and fecal samples, we identified four metabolites for further investigation (VIP > 1, *p* < 0.05, Table 1).

**TABLE 1:** Metabolites significantly increased in both retinal and fecal samples.

ID	Name	Formula	Pubchem	RT (min)	VIP (feces/retina)	*p* (feces/retina)	FC (feces/retina)
1	16‐B1‐Phytop	C15H10O4	50,906,754	5.99	14.568/1.277	7.12E‐4/0.044	45.683/2.481E5
2	4‐Trimethylammoniobutanoic Acid	C7H15NO2	725	0.71	7.126/1.940	0.035/0.003	1.944/1.514
3	Nonadecylic Acid	C19H38O2	12,591	9.31	2.205/2.624	0.004/4.0E‐4	2.607/5.529
4	Indoleacrylic Acid	C11H9NO2	5,375,048	3.67	7.480/3.949	1.07E‐4/0.003	1.642/2.058

Abbreviations: FC, fold change; RT, retention time; VIP, variable importance in projection.

IDA inhibited ferroptosis in a dose‐dependent manner in PC12 cells (Figure 3e), with significant increases in viability at 1 μM (30.08 ± 5.67 vs. 39.80 ± 7.19, *p* < 0.05), 10 μM (30.08 ± 5.67 vs. 80.27 ± 7.38, *p* < 0.001), and 100 μM (30.08 ± 5.67 vs. 75.12 ± 6.59, *p* < 0.001). Nonadecylic acid (NA), 16‐B1‐phytopropanol (16‐B1P), and 4‐trimethylammoniobutanoic acid (TBA) showed no significant protection (all *p* > 0.05, Figure 3f–h).

In a DPPH absorption assay (Figure 3i), IDA (0.01 μM to 100 μM) did not significantly reduce DPPH absorption compared to the control (*p* > 0.05). Fer‐1 significantly reduced DPPH absorption (0.99 ± 0.04 vs. 0.24 ± 0.01, *p* < 0.001).

Western blot analysis of ferroptosis‐related proteins showed no significant changes in Nrf2, NQO1, or SLC7A11 protein levels in PC12 cells treated with RSL3 (Figure S2a–d). However, nAhR levels were significantly increased in the 1 μM (0.40 ± 0.01 vs. 0.19 ± 0.05, *p* < 0.01), 10 μM (0.72 ± 0.04 vs. 0.19 ± 0.05, *p* < 0.01), and 100 μM (0.76 ± 0.06 vs. 0.19 ± 0.05, *p* < 0.01) IDA groups (Figure 3j,k).

Metabolomic analysis revealed a significant reduction in retinal IDA levels in the ONC + DHF + Abx group compared to the ONC + DHF group (Figure 3l). Pathway enrichment analysis (Figure 3m) showed upregulation of ferroptosis‐associated pathways in the ONC + DHF + Abx group.

### 3.3 IDA Protects Retinal Neurons and Promotes Axonal Regeneration by Inhibiting Ferroptosis via the AHR‐ALDH1A3‐FSP1 Axis

**FIGURE 4:**
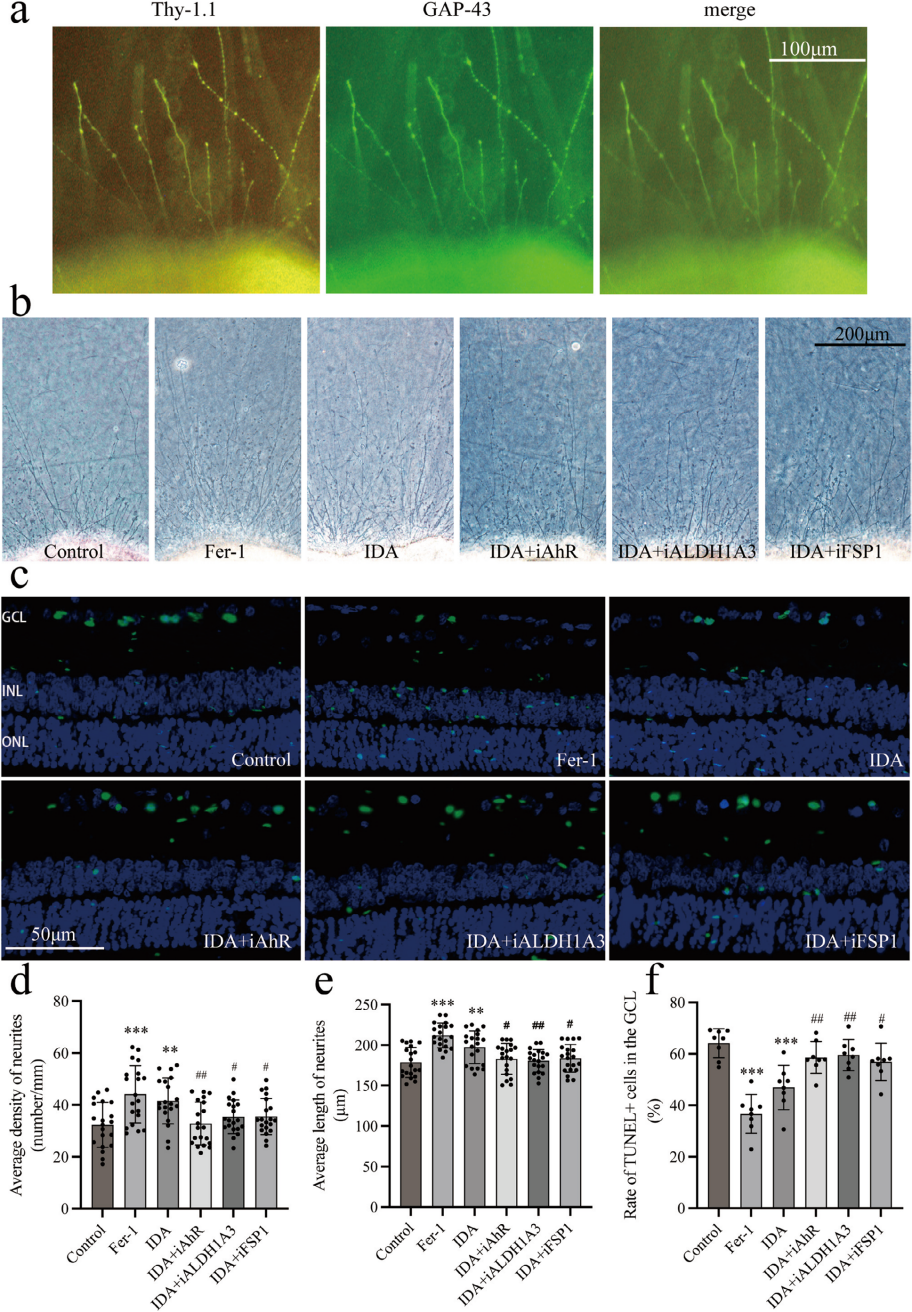
Effects of IDA on neurite regeneration and apoptosis in retinal explants. (a) Immunofluorescence of Thy1.1 and GAP‐43 showing regenerating axons. Scale bar: 100 μm. (b) Light microscopy images of regenerating neurites. Scale bar: 200 μm. (c) TUNEL staining of GCL apoptotic cells with DAPI counterstain. Scale bar: 50 μm. (d) Quantification of regenerating neurite density (number/mm). IDA and Fer‐1 significantly increased neurite density compared to the control, while AhR, ALDH1A3, and FSP1 inhibitors reduced it. (e) Quantification of regenerating neurite length (μm). IDA and Fer‐1 increased neurite length, which was reduced by inhibitors compared to IDA alone. (f) Quantification of TUNEL‐positive cells (%). IDA and Fer‐1 reduced TUNEL‐positive cells, with inhibitors partially reversing this effect. Data are mean ± SEM. Unpaired *t*‐test was used except for IDA + iAhR vs. IDA in (d), and Control vs. IDA or Fer‐1 in (e), where Mann–Whitney *U* test was applied; *n* = 20 for (d, e), *n* = 8 for (f), **p < 0.01, ***p < 0.001 vs. control; #*p* < 0.05, ##*p* < 0.01, ###*p* < 0.01 vs. IDA group.

Cone‐shaped protrusions in retinal explants co‐localized with GAP‐43 and Thy1.1 (Figure 4a), indicating regenerated axons. Both IDA and Fer‐1 groups had significantly higher neurite density than controls (32.29 ± 8.64 vs. 41.56 ± 8.85, *p* < 0.01 for IDA; 32.29 ± 8.64 vs. 44.13 ± 11.07, *p* < 0.001 for Fer‐1; Figure 4b,d) and greater neurite length (178.61 ± 18.43 vs. 211.88 ± 15.26, *p* < 0.001 for IDA; 178.61 ± 18.43 vs. 197.43 ± 20.22 for Fer‐1, *p* < 0.001; Figure 4b,e). Combining IDA with specific inhibitors reduced density and length compared to IDA alone: iAhR (density 41.56 ± 8.85 vs. 32.81 ± 8.30, *p* < 0.01; length 197.43 ± 20.22 vs. 183.10 ± 19.07, *p* < 0.05), iALDH1A3 (density 41.56 ± 8.85 vs. 35.41 ± 6.53, *p* < 0.05; length 197.43 ± 20.22 vs. 180.65 ± 13.99, *p* < 0.01), and iFSP1 (density 41.56 ± 8.85 vs. 35.55 ± 6.98, *p* < 0.05; length 197.43 ± 20.22 vs. 183.62 ± 17.14, *p* < 0.05).

Representative retinal cryosection images (Figure 4c) show significant reductions in TUNEL‐positive cells in Fer‐1 and IDA groups versus controls (64.14 ± 5.66 vs. 36.65 ± 7.54, *p* < 0.001 for Fer‐1; 64.14 ± 5.66 vs. 46.96 ± 8.63, *p* < 0.001 for IDA; Figure 4f). The protective effect of IDA was reversed by inhibitors, with increased TUNEL‐positive cells in IDA + iAhR (46.96 ± 8.63 vs. 58.58 ± 6.16, *p* < 0.01), IDA + iALDH1A3 (46.96 ± 8.63 vs. 59.54 ± 6.07, *p* < 0.01), and IDA + iFSP1 (46.96 ± 8.63 vs. 56.90 ± 7.24, *p* < 0.05).

**FIGURE 5:**
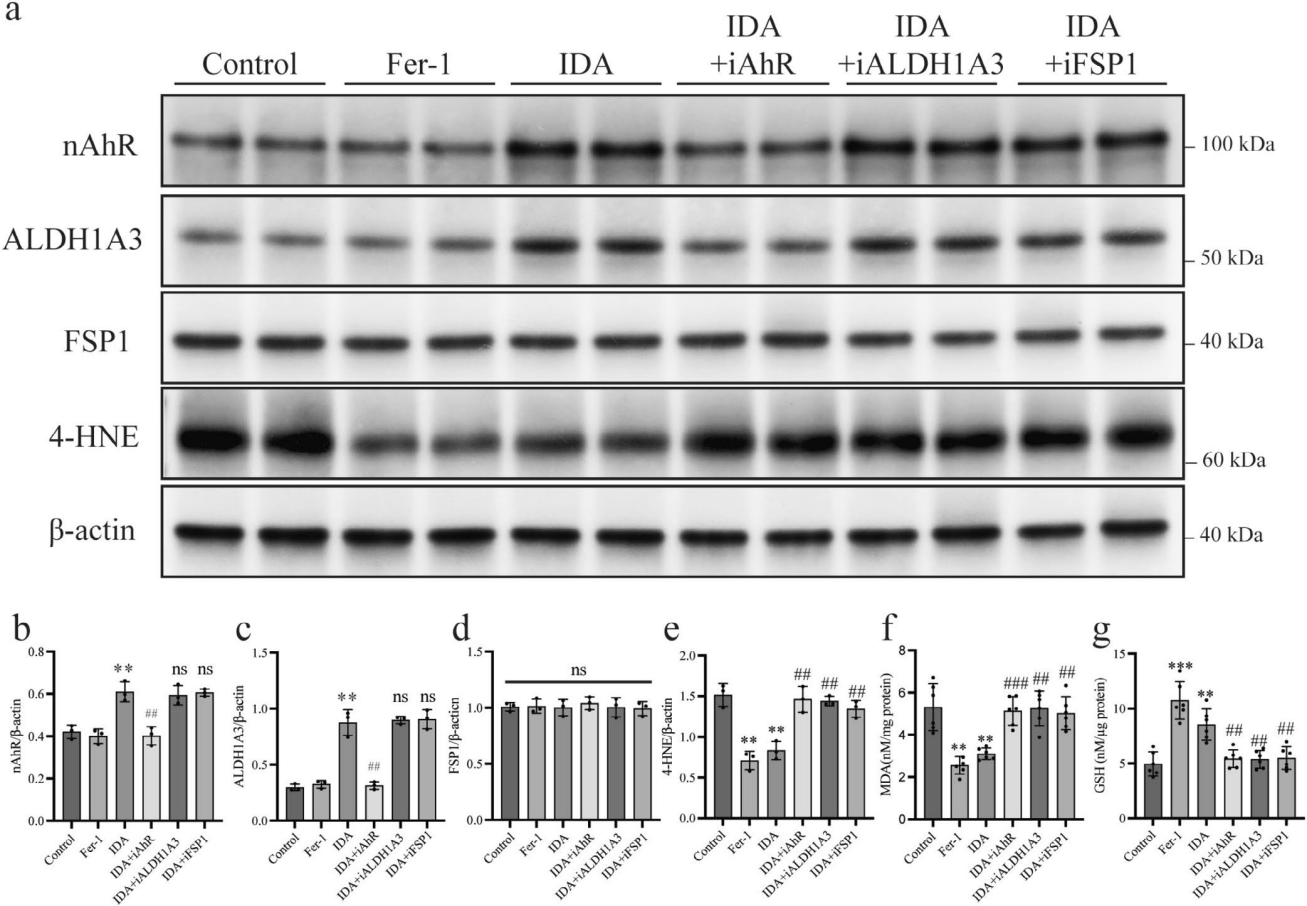
IDA inhibits ferroptosis in retinal neurons via the AhR‐ALDH1A3‐FSP1 pathway. (a) Representative western blot images of f AhR‐ALDH1A3‐FSP1 pathway proteins. (b–d) Quantification of nAhR, ALDH1A3, and FSP1 levels relative to β‐actin. IDA increased nAhR and ALDH1A3 levels, effects partially reversed by the AhR inhibitor (iAhR) but unaffected by ALDH1A3 or FSP1 inhibitors. FSP1 levels remained unchanged. (e–g) Quantification of oxidative stress markers. IDA reduced 4‐HNE and MDA levels and increased GSH levels, effects negated by iAhR, iALDH1A3, or iFSP1. Data are mean ± SEM (*n* = 3). One‐way ANOVA with Tukey's post hoc test for (d); unpaired *t*‐test for (b, c, e–g). *p* values were adjusted for multiple testing by the Benjamini Hochberg method. **p < 0.01, ***p < 0.001 vs. control; ##*p* < 0.01, ###*p* < 0.001 vs. IDA group; ns (above bars), *p* > 0.05 vs. ONC + DHF; ns (above horizontal lines), *p* > 0.05 among groups below the line.

Western blot analysis (Figure 5a) showed that IDA significantly increased nAhR and ALDH1A3 levels compared to controls: nAhR from 0.42 ± 0.03 to 0.61 ± 0.05 (BH‐adjusted *p* < 0.01; Figure 5b) and ALDH1A3 from 0.30 ± 0.03 to 0.88 ± 0.11 (BH‐adjusted *p* < 0.01; Figure 5c). AhR inhibitor (iAhR) reversed the upregulation of nAhR (0.61 ± 0.05 vs. 0.40 ± 0.04, BH‐adjusted *p* < 0.01; Figure 5b) and ALDH1A3 (0.88 ± 0.11 vs. 0.31 ± 0.03, BH‐adjusted *p* < 0.01; Figure 5c). FSP1 expression and other inhibitors did not affect nAhR or ALDH1A3 levels (BH‐adjusted *p* > 0.05; Figure 5d). IDA treatment did not significantly alter the expression of ferroptosis‐related proteins GPX4, ACSL4, SLC7A11, GCH1, FTH1, or DHODH (Figure S3a–g).

IDA significantly reduced lipid peroxidation products 4‐HNE (1.52 ± 0.14 vs. 0.83 ± 0.11, BH‐adjusted *p* < 0.01; Figure 5e) and MDA (5.31 ± 1.11 vs. 3.10 ± 0.27, BH‐adjusted *p* < 0.01; Figure 5f), and increased GSH levels (4.96 ± 1.10 vs. 8.54 ± 1.43, BH‐adjusted *p* < 0.01; Figure 5g) compared to controls. These effects were reversed by inhibitors: iAhR (4‐HNE: BH‐adjusted *p* < 0.01; MDA: BH‐adjusted *p* < 0.001; GSH: BH‐adjusted *p* < 0.01; Figure 5e–g), iALDH1A3 (4‐HNE: BH‐adjusted *p* < 0.01; MDA: BH‐adjusted *p* < 0.01; GSH: BH‐adjusted *p* < 0.01; Figure 5e–g), and iFSP1 (4‐HNE: BH‐adjusted *p* < 0.01; MDA: BH‐adjusted *p* < 0.01; GSH: BH‐adjusted *p* < 0.001 for iAhR; Figure 5e–g).

### 3.4 7,8‐DHF Protects RGCs and Their Function by Inhibiting Ferroptosis via the IDA‐AhR‐ALDH1A3‐FSP1 Axis

**FIGURE 6:**
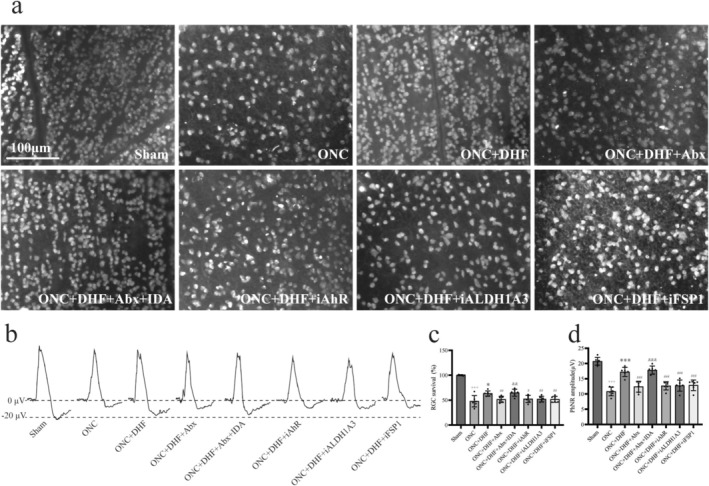
Neuroprotective effects of 7,8‐DHF and the gut microbiota‐IDA‐AhR‐ALDH1A3‐FSP1 pathway on RGC survival and retinal function following ONC injury. (a) Retinal flat‐mount images of RBPMS‐labeled RGCs. Scale bar: 100 μm. (b) Representative PhNR traces showing retinal function. (c) Quantification of RGC survival (%). RGC density was reduced in the ONC group compared to the Sham group, partially preserved by 7,8‐DHF, attenuated by antibiotics, and restored by IDA. AhR, ALDH1A3, and FSP1 inhibitors partially reduced RGC survival. (d) Quantification of PhNR amplitudes (μV). Retinal function decreased in the ONC group, was partially restored by 7,8‐DHF, diminished by antibiotics, and recovered by IDA. AhR, ALDH1A3, and FSP1 inhibitors partially reduced the recovery. Data are mean ± SD (*n* = 6). Unpaired *t*‐test was used except for Sham vs. ONC in (c), where Welch's *t*‐test was applied; +++*p* < 0.001 vs. Sham; *p < 0.05, ***p < 0.001 vs. ONC; #*p* < 0.05, ##*p* < 0.01, ###*p* < 0.001 vs. ONC + DHF; &&*p* < 0.01, &&&*p* < 0.001 vs. ONC + DHF + Abx.

The Sham group had near 100% survival, while the ONC group showed a dramatic reduction (99.97 ± 1.04 vs. 47.83 ± 11.38, *p* < 0.001, Figure 6a,c). 7,8‐DHF treatment partially restored RGC survival (47.83 ± 11.38 vs. 63.26 ± 5.28, *p* < 0.05, Figure 6a,c), but antibiotics (ONC + DHF + Abx group) reversed this benefit (63.26 ± 5.28 vs. 51.59 ± 6.25, *p* < 0.01, Figure 6a,c). Supplementing with IDA (ONC + DHF + Abx + IDA group) restored survival (64.83 ± 7.07 vs. 51.59 ± 6.25, *p* < 0.01, Figure 6a,c). Inhibiting AhR, ALDH1A3, and FSP1 reduced survival (63.26 ± 5.28 vs. 52.42 ± 7.14, *p* < 0.05 for iAhR; 63.26 ± 5.28 vs. 52.01 ± 7.75, *p* < 0.01 for iALDH1A3; 63.26 ± 5.28 vs. 51.66 ± 6.00, *p* < 0.01 for iFSP1, Figure 6a,c).

The Sham group had the highest amplitude, while the ONC group showed a significant reduction (20.70 ± 1.30 vs. 10.85 ± 1.44, *p* < 0.001; Figure 6b,d). The ONC + DHF group exhibited partial recovery (17.23 ± 1.49 vs. 10.85 ± 1.44, *p* < 0.001; Figure 6b,d), but the ONC + DHF + Abx group showed decreased amplitude compared to ONC + DHF (12.38 ± 1.78 vs. 17.23 ± 1.49, *p* < 0.001; Figure 6b,d). IDA treatment restored PhNR amplitude (17.81 ± 1.35 vs. 12.38 ± 1.78, *p* < 0.001; Figure 6b,d). Inhibitors of AhR, ALDH1A3, and FSP1 reduced amplitude compared to DHF treatment (17.23 ± 1.49 vs. 12.67 ± 1.35, *p* < 0.001 for iAhR; 17.23 ± 1.49 vs. 12.74 ± 1.84, *p* < 0.001 for iALDH1A3; 17.23 ± 1.49 vs. 12.70 ± 1.37, *p* < 0.001 for iFSP1; Figure 6b,d).

**FIGURE 7:**
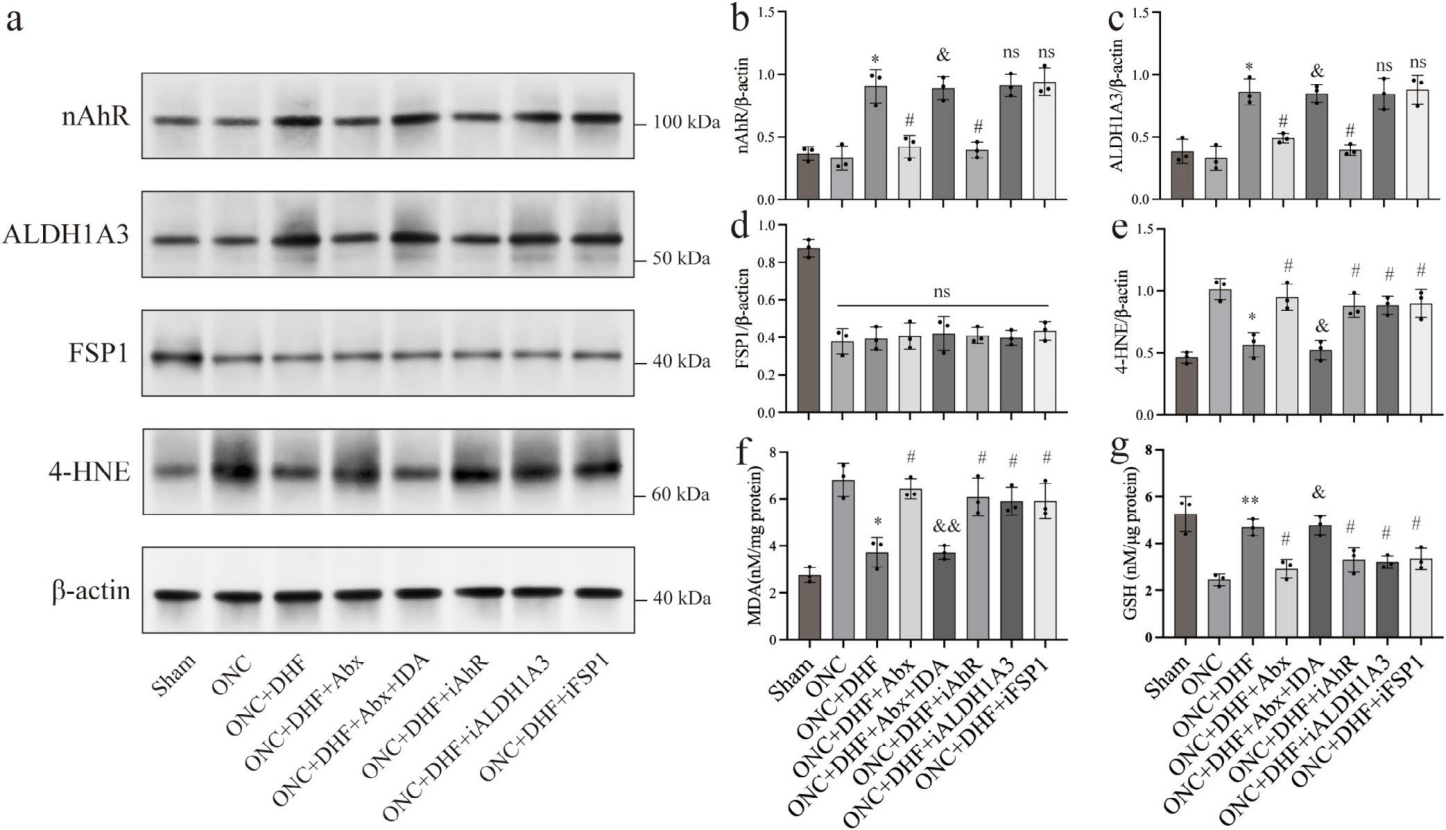
Effects of 7,8‐DHF, gut microbiota disruption, and IDA on AhR‐ALDH1A3‐FSP1 pathway proteins and oxidative stress in retinal tissues following ONC injury. (a) Representative western blot images for AhR‐ALDH1A3‐FSP1 pathway proteins. (b–d) Quantification of nAhR, ALDH1A3, and FSP1 levels relative to β‐actin. 7,8‐DHF increased nAhR and ALDH1A3 levels, which were reduced by antibiotics and restored by IDA. AhR inhibitors decreased nAhR and ALDH1A3, while ALDH1A3 or FSP1 inhibition had no effect on nAhR. FSP1 levels remained unchanged across groups. (e–g) Oxidative stress markers: 7,8‐DHF reduced 4‐HNE and MDA while increasing GSH levels, effects reversed by antibiotics and restored by IDA. Inhibitors of AhR, ALDH1A3, and FSP1 partially reversed these effects, implicating the AhR‐ALDH1A3‐FSP1 pathway. Data are mean ± SEM (*n* = 3). *p* values were adjusted for multiple testing by the Benjamini Hochberg method. *p < 0.05, **p < 0.01 vs. ONC; #*p* < 0.05 vs. ONC + DHF; &*p* < 0.05, &&*p* < 0.01 vs. ONC + DHF + Abx; ns (above bars), *p* > 0.05 vs. ONC + DHF; ns (above horizontal lines), *p* > 0.05 among groups below the line.

Western blotting revealed that nAhR and ALDH1A3 levels were significantly higher in the ONC + DHF group than in the ONC group (0.91 ± 0.13 vs. 0.33 ± 0.10, BH‐adjusted *p* < 0.05 for nAhR; 0.86 ± 0.10 vs. 0.33 ± 0.10, BH‐adjusted *p* < 0.05 for ALDH1A3; Figure 7a–c). The ONC + DHF + Abx group showed reduced nAhR (0.91 ± 0.13 vs. 0.42 ± 0.09, BH‐adjusted *p* < 0.05; Figure 7a,b) and ALDH1A3 (0.86 ± 0.10 vs. 0.49 ± 0.04, BH‐adjusted *p* < 0.05; Figure 7a,c). IDA supplementation restored nAhR (0.89 ± 0.09 vs. 0.42 ± 0.09, BH‐adjusted *p* < 0.05; Figure 7a,b) and ALDH1A3 (0.85 ± 0.07 vs. 0.49 ± 0.04, BH‐adjusted *p* < 0.05; Figure 7a,c). AhR inhibition (ONC + DHF + iAhR) decreased both nAhR (0.91 ± 0.13 vs. 0.40 ± 0.06, BH‐adjusted *p* < 0.05; Figure 7a,b) and ALDH1A3 (0.86 ± 0.10 vs. 0.40 ± 0.04, BH‐adjusted *p* < 0.05; Figure 7a,c). In contrast, inhibition of ALDH1A3 or FSP1 did not significantly affect nAhR or ALDH1A3 levels (iALDH1A3: 0.91 ± 0.13 vs. 0.91 ± 0.09, BH‐adjusted *p* > 0.05 for nAhR; 0.86 ± 0.10 vs. 0.85 ± 0.12, BH‐adjusted *p* > 0.05 for ALDH1A3; iFSP: 0.91 ± 0.13 vs. 0.94 ± 0.11, BH‐adjusted *p* > 0.05 for nAhR; 0.86 ± 0.10 vs. 0.88 ± 0.11, BH‐adjusted *p* > 0.05 for ALDH1A3; Figure 7a–c). No treatments altered retinal FSP1 protein levels (*F* = 0.261, BH‐adjusted *p* > 0.05; Figure 7d). No significant changes were observed in GPX4, ACSL4, SLC7A11, GCH1, FTH1, or DHODH protein levels among the ONC and treatment groups (Figure S4a–g).

Oxidative stress analysis revealed decreased 4‐HNE (1.01 ± 0.08 vs. 0.56 ± 0.10, BH‐adjusted *p* < 0.05; Figure 7e) and MDA (6.81 ± 0.71 vs. 3.73 ± 0.62, BH‐adjusted *p* < 0.05; Figure 7f) levels alongside increased GSH (2.46 ± 0.25 vs. 4.69 ± 0.35, BH‐adjusted *p* < 0.01; Figure 7g) levels in the ONC + DHF group compared to the ONC group. These effects were reversed in the ONC + DHF + Abx group (0.95 ± 0.11 vs. 0.56 ± 0.10, BH‐adjusted *p* < 0.05 for 4‐HNE; 6.57 ± 0.36 vs. 3.73 ± 0.62, BH‐adjusted *p* < 0.05 for MDA; 2.92 ± 0.40 vs. 4.69 ± 0.35, BH‐adjusted *p* < 0.05 for GSH; Figure 7e–g) due to gut microbiota disruption but were restored by IDA in the ONC + DHF + Abx + IDA group (0.95 ± 0.11 vs. 0.52 ± 0.08, BH‐adjusted *p* < 0.05 for 4‐HNE; 6.44 ± 0.42 vs. 3.72 ± 0.29, BH‐adjusted *p* < 0.01 for MDA; 2.92 ± 0.40 vs. 4.69 ± 0.35, BH‐adjusted *p* < 0.05 for GSH; Figure 7e–g). Inhibitors of AhR, ALDH1A3, or FSP1 elevated 4‐HNE (0.88 ± 0.09 vs. 0.56 ± 0.10, BH‐adjusted *p* < 0.05 for iAhR; 0.88 ± 0.07 vs. 0.56 ± 0.10, BH‐adjusted *p* < 0.05 for iALDH1A3; 0.90 ± 0.11 vs. 0.56 ± 0.10, BH‐adjusted *p* < 0.05 for iFSP1; Figure 7e) and MDA (6.09 ± 0.80 vs. 3.73 ± 0.62, BH‐adjusted *p* < 0.05 for iAhR; 5.91 ± 0.59 vs. 3.73 ± 0.62, BH‐adjusted *p* < 0.05 for iALDH1A3; 5.92 ± 0.76 vs. 3.73 ± 0.62, BH‐adjusted *p* < 0.05 for iFSP1; Figure 7f) levels while lowering GSH (3.30 ± 0.51 vs. 4.69 ± 0.35, BH‐adjusted *p* < 0.05 for iAhR; 3.21 ± 0.27 vs. 4.69 ± 0.35, BH‐adjusted *p* < 0.05 for iALDH1A3; 3.34 ± 0.46 vs. 4.69 ± 0.35, BH‐adjusted *p* < 0.05 for iFSP1; Figure 7g) levels.

### 3.5 7,8‐DHF May Regulate Gut IDA Levels by Modulating the Parasutterella Microbiome

**FIGURE 8:**
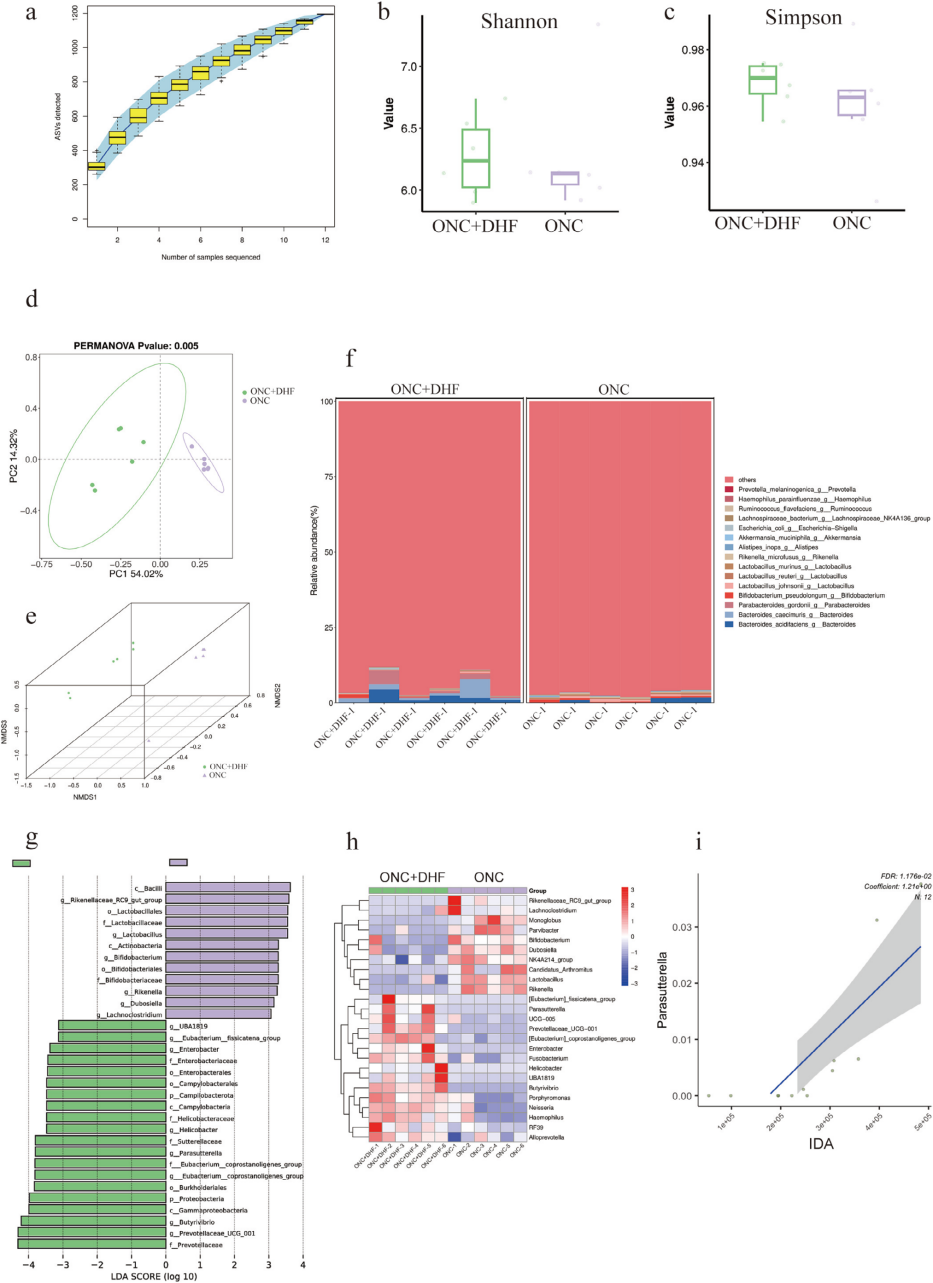
Effects of 7,8‐DHF on gut microbiota composition and its correlation with IDA levels in ONC mice. (a) Rarefaction curves for observed amplicon sequence variants in fecal samples, indicating sufficient sequencing depth. (b, c) Alpha diversity indices (Shannon and Simpson) showing no significant differences in richness or evenness between the ONC and ONC + DHF groups. (d, e) β diversity analysis via PCoA and NMDS plots based on Bray‐Curtis distances, revealing significant differences in microbial community composition between the ONC and ONC + DHF groups (PERMANOVA *p* = 0.005). (f) Bar chart of bacterial genera abundance, demonstrating that 7,8‐DHF treatment markedly altered gut microbiota composition. (g) LEfSe analysis (LDA score > 3.0, *p* < 0.05) showing significantly increased genera (e.g., UBA1819, Eubacterium_fissicatena_group, Butyrivibrio) and decreased genera (e.g., Rikenellaceae_RC9_gut_group, Lactobacillus) in the ONC +7,8‐DHF group compared to ONC. (h) Heatmap of differentially abundant genera validated by Wilcoxon test (*p* < 0.05), with warmer colors indicating higher abundance. (i) MaAsLin analysis showing a significant positive correlation between Butyrivibrio abundance and gut IDA levels (FDR < 0.05, Coefficient = 6.93). The regression line (blue) and shaded area represent the trend and confidence interval, respectively. Unpaired *t*‐test for (b, c), Wilcoxon test for (h). *n* = 6 for all panels.

Rarefaction curves (Figure 8a) showed species accumulation curves reaching saturation, indicating sufficient sequencing depth. Alpha diversity analysis revealed no significant differences in Shannon and Simpson indices between the ONC + DHF and ONC groups (*p* > 0.05; Figure 8b,c). Beta diversity analyses, including PCoA (Figure 8d) and NMDS (Figure 8e) based on Bray‐Curtis distance, showed significant separation between the 7,8‐DHF‐treated and control groups, confirmed by Adonis analysis (*R* ^2^ = 0.50091, *p* < 0.01). The community structure bar chart (Figure 8f) displayed clear differences.

LEfSe analysis identified eight genera with increased abundance in the ONC + DHF group (LDA score > 3.0, *p* < 0.05; Figure 8g), including UBA1819, Eubacterium_fissicatena_group, Enterobacter, Helicobacter, Parasutterella, Eubacterium_coprostanoligenes_group, Butyrivibrio, and Prevotellaceae_UCG_001, and six genera with decreased abundance, including Rikenellaceae_RC9_gut_group, Lactobacillus, Bifidobacterium, Rikenella, Dubosiella, and Lachnoclostridium, confirmed by the Wilcoxon test (*p* < 0.05; Figure 8h).

MaAsLin analysis revealed a significant positive correlation between Parasutterella and gut IDA levels (FDR < 0.05, Coefficient: 1.21; Figure 8i).

## 4 Discussion

In this study, we have demonstrated that oral administration of 7,8‐DHF exerts significant neuroprotective effects on RGCs in vivo and in vitro models. The protective mechanism is primarily mediated through the modulation of the gut‐retina axis, leading to the inhibition of ferroptosis via the IDA‐AhR‐ALDH1A3‐FSP1 pathway. Our findings reveal that 7,8‐DHF enhances RGC survival and retinal function by increasing the levels of IDA, a gut‐derived metabolite, which in turn activates the AhR‐ALDH1A3‐FSP1 axis in the retina. This pathway effectively inhibits ferroptosis, as evidenced by reduced oxidative stress markers, enhanced antioxidant capacity, and preserved mitochondrial integrity.

7,8‐DHF, a flavonoid compound identified as a TrkB receptor agonist, activates the TrkB pathway to enhance neuronal survival, synaptic plasticity, and regeneration through PI3K/AKT, MAPK/ERK, and PLCγ signaling [5, 9, 10, 11]. Preclinical studies demonstrate its potential to reduce neurodegeneration and improve recovery from CNS diseases [12, 13]. However, its clinical use is limited by pharmacokinetic issues. Intravenous administration causes systemic toxicity, while intravitreal injection poses significant ocular risks, including hemorrhage and infection. Oral administration is safer but results in low plasma concentrations due to rapid metabolism and poor absorption [4], preventing effective retinal neuroprotection. These challenges necessitate alternative strategies to achieve therapeutic retinal concentrations with minimal adverse effects.

Our study shows that two‐week oral 7,8‐DHF pre‐ONC significantly protects mouse RGCs by modulating ferroptosis via the gut‐retina axis, which is part of gut‐brain communication and plays a vital role in retinal health [14, 15, 16]. Studies focus on how gut microbiota and metabolites, like short‐chain fatty acids [17, 18] and bile acids [19, 20], influence retinal inflammation [21], oxidative stress [22], and neurodegeneration [23], highlighting gut‐derived signals' role in retinal pathology. This suggests the gut‐retina axis as a target for neurodegenerative retinal diseases. This study introduces IDA's role in retinal neuroprotection, inhibiting ferroptosis in RGCs, preserving mitochondrial function, and improving cell survival. However, elevated gut IDA may also impact other areas. Recent findings show ferroptosis inhibition might accelerate cancer [24, 25, 26], especially in gastrointestinal cancers [25], necessitating further research into the balance between neuroprotective and tumor‐promoting effects.

To identify microbial contributors to gut‐derived IDA, microbiome shifts under 7,8‐DHF treatment were examined in this study. Parasutterella, a commensal genus linked to intestinal homeostasis and bile acid metabolism [27, 28], correlated positively with IDA levels, suggesting involvement in gut‐retina axis modulation. Beyond individual taxa, these alterations imply that short‐term treatment may induce lasting neuroprotective effects via microbiome‐mediated mechanisms. Although our study assessed microbial changes over a 4‐week treatment window (2 weeks before to 2 weeks after ONC), we did not evaluate their persistence post‐treatment. Nonetheless, the observed remodeling indicates potential for sustained metabolic reprogramming. This is supported by evidence that transient microbial interventions can drive long‐term functional shifts through ecological succession and host–microbiome interactions [29]. Thus, short‐term preconditioning with 7,8‐DHF may establish self‐sustaining microbial–metabolite networks with lasting retinal benefits. Future studies should clarify the durability and mechanistic basis of these effects and assess pulse‐dosing strategies to reduce chronic exposure while maintaining efficacy.

Furthermore, 7,8‐DHF influences other gut microbiota discovered by this study, such as Enterobacter and Helicobacter, which may modulate immune and metabolic pathways contributing to systemic health [30]. Changes in Butyrivibrio and Prevotellaceae genera, known for their role in short‐chain fatty acid (SCFA) production [31, 32], may enhance anti‐inflammatory effects both locally and systemically. In conclusion, the microbiota‐driven changes observed following oral 7,8‐DHF administration indicate that its benefits likely extend beyond retinal protection, with the potential to influence additional physiological functions. Further investigation is needed to explore these broader effects and fully understand the systemic benefits of 7,8‐DHF.

Ferroptosis is iron‐dependent cell death driven by phospholipid peroxidation, regulated by proteins like GPX4, FSP1, the BH4 system, and DHODH, which convert lipid hydroperoxides to non‐toxic lipid alcohols, preventing ferroptotic death [33, 34]. In our study, IDA activates AhR to upregulate ALDH1A3, enhancing FSP1‐driven CoQ10 reduction to prevent RGC ferroptosis via antioxidant mechanisms. While the observed decreases in MDA and 4‐HNE levels, along with elevated GSH, support ferroptosis inhibition, the absence of complementary assays—such as labile iron quantification and dynamic lipid peroxidation tracking (e.g., C11‐BODIPY staining)—limits the spatiotemporal resolution of ferroptosis assessment. Future studies incorporating iron chelation models and real‐time oxidative stress imaging will be essential to delineate ferroptosis regulation in retinal pathology.

The translational relevance of our findings merits further discussion. Unlike conventional neuroprotectants that depend on systemic bioavailability, 7,8‐DHF may exert therapeutic effects via gut‐localized mechanisms, potentially decoupling efficacy from plasma drug levels. This shift suggests that human dosing could focus on modulating microbial ecology rather than relying on traditional body surface area‐based scaling. Notably, the induction of microbial metabolites such as IDA likely follows saturable, host‐microbiota‐regulated kinetics rather than linear dose–response relationships. The limited intestinal absorption of 7,8‐DHF, often considered a pharmacokinetic drawback, may instead confer therapeutic specificity by concentrating its action within the gut while minimizing systemic TrkB activation. For long‐term clinical application, it remains important to monitor systemic iron homeostasis, given the fundamental roles of ferroptosis and redox balance in multiple organ systems.

## 5 Conclusion

Our findings indicate that 7,8‐DHF alters gut microbiota by increasing Parasutterella, resulting in higher IDA levels. This enhances retinal neuroprotection via the AhR‐ALDH1A3‐FSP1 pathway in RGCs. Understanding these microbial shifts and their impact on IDA production is key for developing targeted gut‐retina axis therapies for neuroprotection.

## Author Contributions

Yanping Zhou, Yifan Feng, Yingxi Zhao, Yu Wu, Min Li, Xi Yang, and Xinyuan Wu conducted the gathering, examination, and understanding of data. Xiangwu Chen proposed the idea for the research and carried out the investigation. The article was written by Yanping Zhou, Yifan Feng, and Xiangwu Chen. The final article was read and approved by all authors.

## Disclosure

*Declaration of Generative AI and AI‐Assisted Technologies in the Writing Process*: During the preparation of this work, the authors utilized ChatGPT to improve both readability and language quality. Following the application of this tool, the authors conducted a thorough review and made necessary edits, thereby assuming full responsibility for the final content of the publication.

## Conflicts of Interest

The authors declare no conflicts of interest.

## Supporting information


**Figure S1.** Metabolomic alterations in fecal and retinal samples induced by 7,8‐DHF following ONC injury. (a, b) OPLS‐DA score plots of fecal (a) and retinal (b) metabolomics showing distinct metabolic profiles between the ONC and ONC +7,8‐DHF groups. (c, d) Volcano plots of differential metabolites in fecal (c) and retinal (d) samples. Upregulated (red) and downregulated (blue) metabolites were identified based on VIP > 1 and *p* < 0.05. In fecal samples, 218 metabolites were upregulated and 173 downregulated. In retinal samples, 117 metabolites were upregulated and 51 downregulated. Data are presented as mean ± SEM; *n* = 6 for all panels.

**Figure S2.** Effects of IDA on ferroptosis proteins in PC12 cells. (a)Western blot analysis of ferroptosis‐related proteins in PC12 cells treated with RSL3 and IDA (0–100 μM). (b–d) Quantification of Nrf2, NQO1, and SLC7A11 protein levels. IDA did not change the levels of Nrf2, NQO1, or SLC7A11 at 1, 10, and 100 μM. Data are mean ± SEM (*n* = 3). One‐way ANOVA with Tukey’s post hoc test for all panels. ns (above horizontal lines), *p* > 0.05 among groups below the line.

**Figure S3.** Effects of IDA on ferroptosis‐related protein expression. (a) Representative western blot images of ferroptosis‐related proteins. (b–g) Quantification of GPX4, ACSL4, SLC7A11, GCH1, FTH1, and DHODH levels. No changes were observed for these proteins under IDA treatments. Data are mean ± SEM (*n* = 3). Unpaired *t*‐test for (b–d, f). One‐way ANOVA with Tukey’s post hoc test for (e, g). *p* values were adjusted for multiple testing by the Benjamini Hochberg method. *p < 0.05, **p < 0.01 vs. control; ns (above bars), *p* > 0.05 vs. ONC + DHF; ns (above horizontal lines), *p* > 0.05 among groups below the line.

**Figure S4.** Effects of 7,8‐DHF, gut microbiota disruption, and IDA on ferroptosis‐related proteins n retinal tissues following ONC injury. (a) Representative western blot images for ferroptosis‐related proteins. (b–g) Quantification of GPX4, ACSL4, SLC7A11, GCH1, FTH1, and DHODH showed no significant changes, indicating that neuroprotection by 7,8‐DHF and IDA does not involve these proteins. Data are mean ± SEM (*n* = 3). One‐way ANOVA with Tukey’s post hoc test for all panels. ns (above horizontal lines), *p* > 0.05 among groups below the line.

Data S1.

## Data Availability

The datasets used and/or analyzed during the current study are available from the corresponding author on reasonable request.
