# Supplementary figures and images for: Oral 7,8‐Dihydroxyflavone Protects Retinal Ganglion Cells by Modulating the Gut‐Retina Axis and Inhibiting Ferroptosis via the Indoleacrylic Acid‐AhR‐ALDH1A3‐FSP1 Pathway
Source: CNS Neurosci Ther. 2025 May 14;31(5):e70442. doi: 10.1111/cns.70442 (PMC12076127; doi:10.1111/cns.70442)

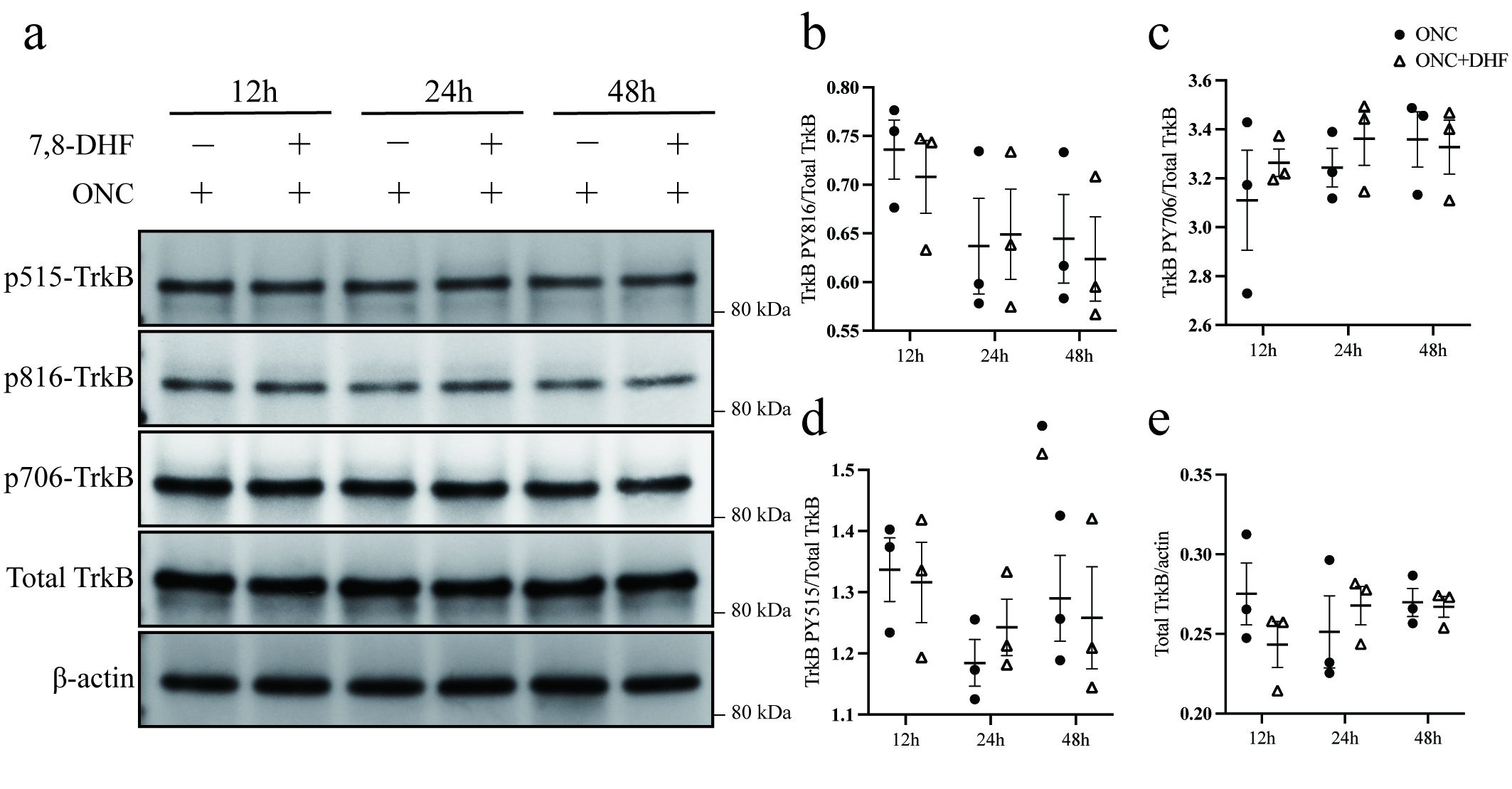

Supplement: Supplementary file 1 — Figure S1. Metabolomic alterations in fecal and retinal samples induced by 7,8‐DHF following ONC injury. (a, b) OPLS‐DA score plots of fecal (a) and retinal (b) metabolomics showing distinct metabolic profiles between the ONC and ONC +7,8‐DHF groups. (c, d) Volcano plots of differential metabolites in fecal (c) and retinal (d) samples. Upregulated (red) and downregulated (blue) metabolites were identified based on VIP > 1 and p < 0.05. In fecal samples, 218 metabolites were upregulated and 173 downregulated. In retinal samples, 117 metabolites were upregulated and 51 downregulated. Data are presented as mean ± SEM; n = 6 for all panels. [file CNS-31-e70442-s005.tif]

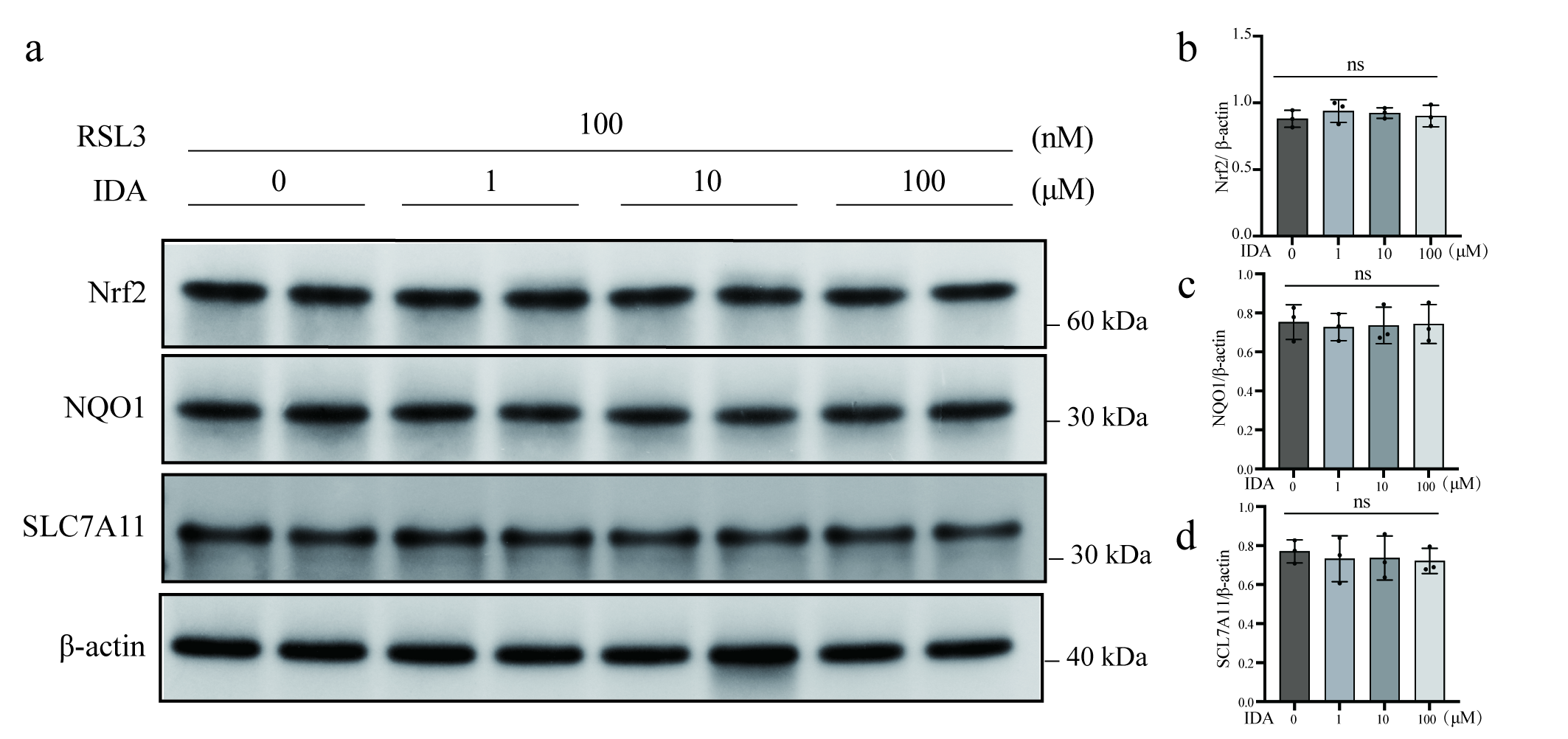

Supplement: Supplementary file 2 — Figure S2. Effects of IDA on ferroptosis proteins in PC12 cells. (a)Western blot analysis of ferroptosis‐related proteins in PC12 cells treated with RSL3 and IDA (0–100 μM). (b–d) Quantification of Nrf2, NQO1, and SLC7A11 protein levels. IDA did not change the levels of Nrf2, NQO1, or SLC7A11 at 1, 10, and 100 μM. Data are mean ± SEM (n = 3). One‐way ANOVA with Tukey’s post hoc test for all panels. ns (above horizontal lines), p > 0.05 among groups below the line. [file CNS-31-e70442-s004.tif]

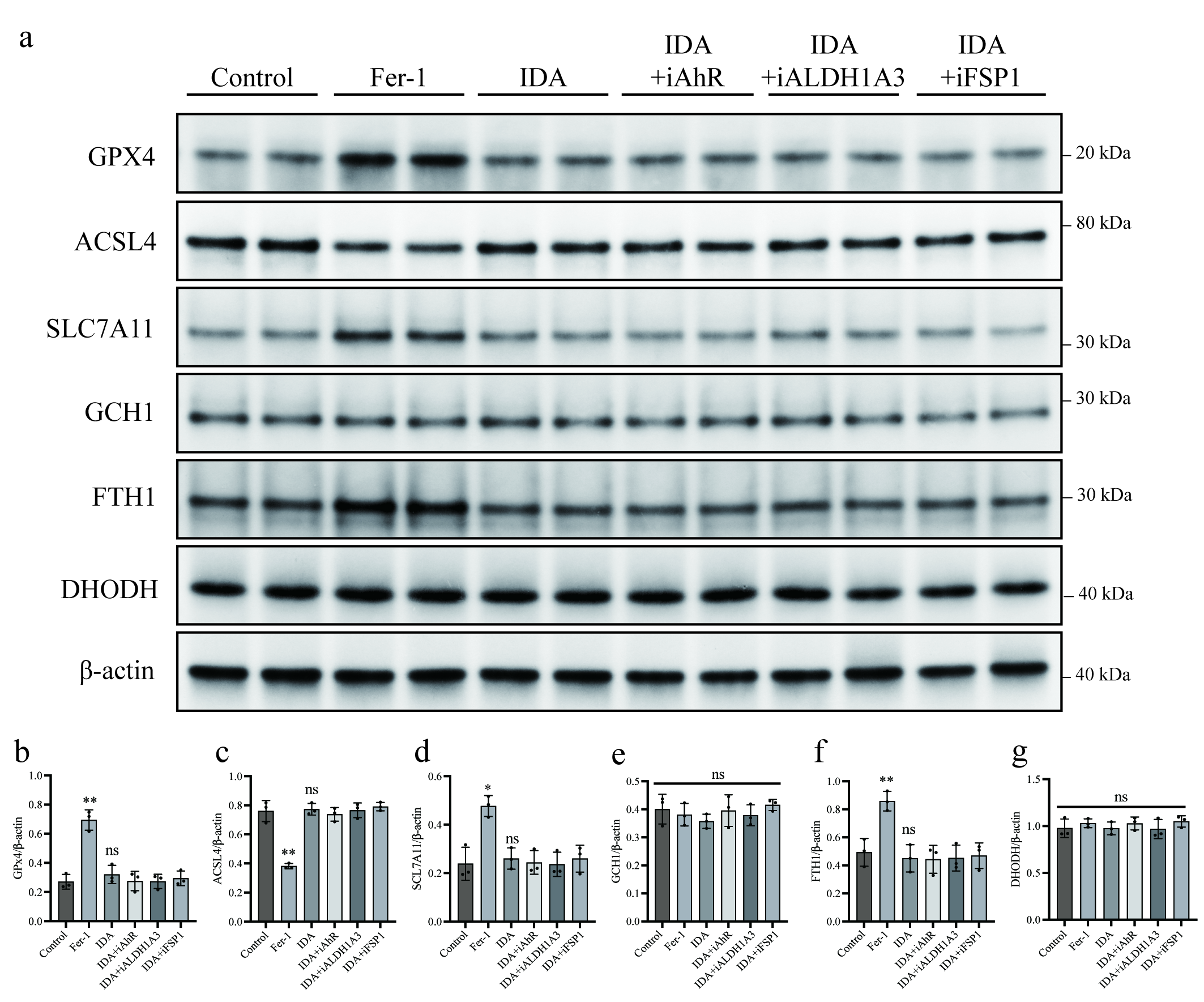

Supplement: Supplementary file 3 — Figure S3. Effects of IDA on ferroptosis‐related protein expression. (a) Representative western blot images of ferroptosis‐related proteins. (b–g) Quantification of GPX4, ACSL4, SLC7A11, GCH1, FTH1, and DHODH levels. No changes were observed for these proteins under IDA treatments. Data are mean ± SEM (n = 3). Unpaired t‐test for (b–d, f). One‐way ANOVA with Tukey’s post hoc test for (e, g). p values were adjusted for multiple testing by the Benjamini Hochberg method. *p < 0.05, **p < 0.01 vs. control; ns (above bars), p > 0.05 vs. ONC + DHF; ns (above horizontal lines), p > 0.05 among groups below the line. [file CNS-31-e70442-s003.tif]

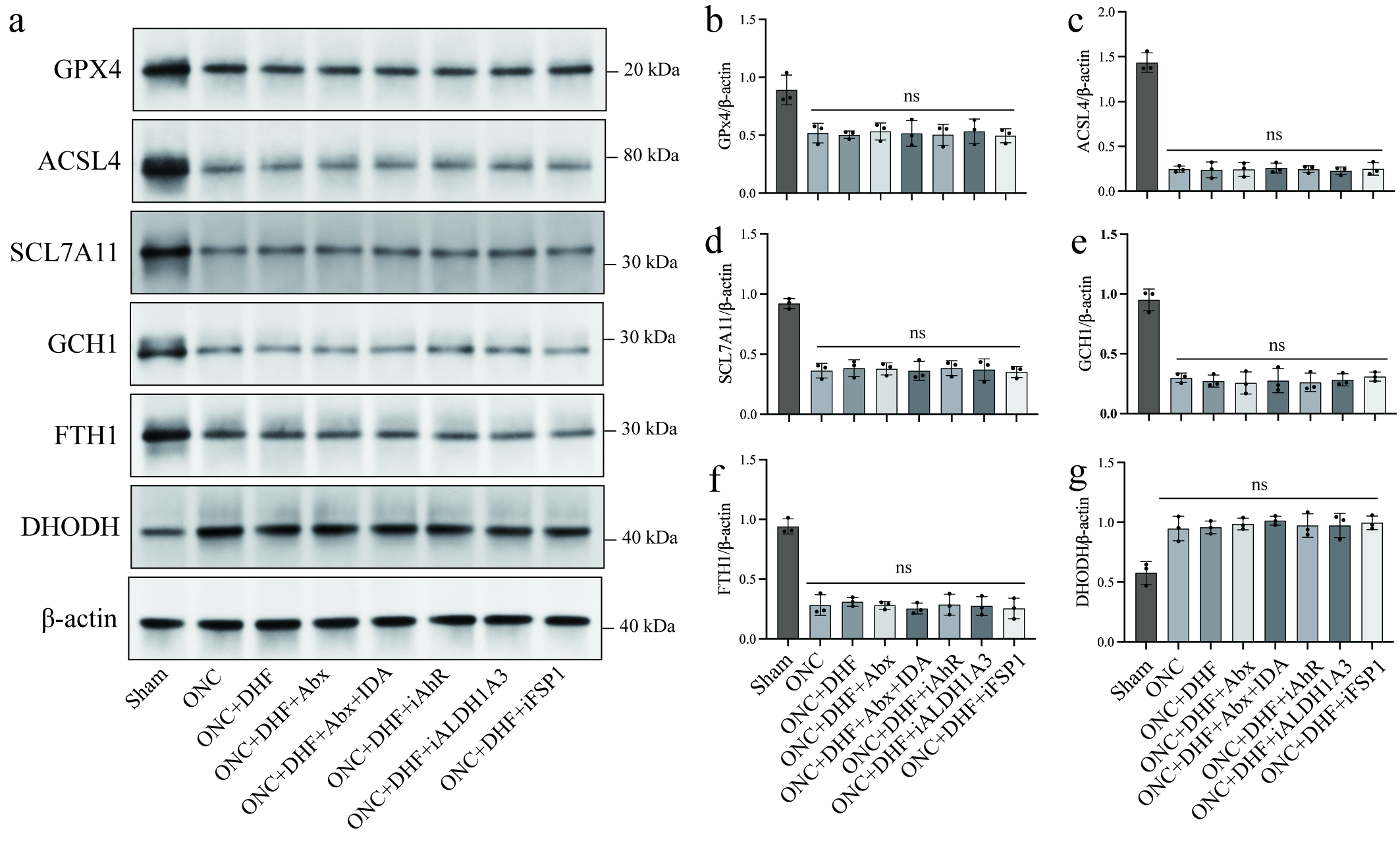

Supplement: Supplementary file 4 — Figure S4. Effects of 7,8‐DHF, gut microbiota disruption, and IDA on ferroptosis‐related proteins n retinal tissues following ONC injury. (a) Representative western blot images for ferroptosis‐related proteins. (b–g) Quantification of GPX4, ACSL4, SLC7A11, GCH1, FTH1, and DHODH showed no significant changes, indicating that neuroprotection by 7,8‐DHF and IDA does not involve these proteins. Data are mean ± SEM (n = 3). One‐way ANOVA with Tukey’s post hoc test for all panels. ns (above horizontal lines), p > 0.05 among groups below the line. [file CNS-31-e70442-s002.tif]

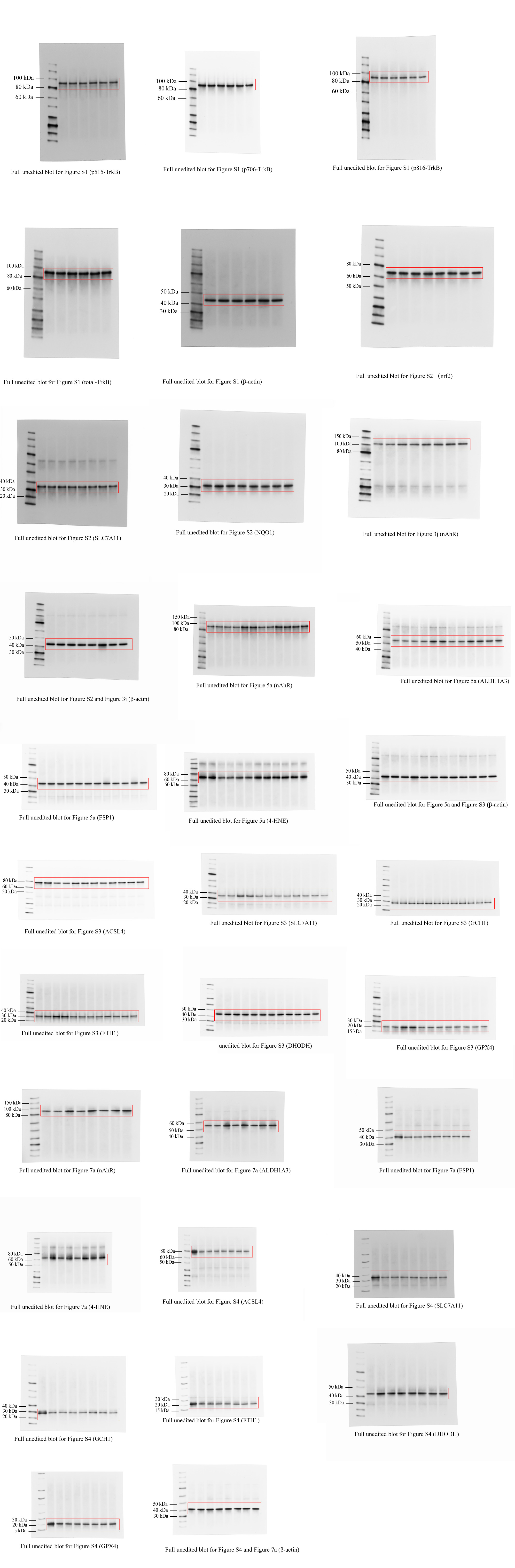

Supplement: Supplementary file 5 — Data S1. [file CNS-31-e70442-s001.jpg]
